# Exhaled nitric oxide and quality of life in asthmatic teenagers

**DOI:** 10.1186/1939-4551-8-S1-A14

**Published:** 2015-04-08

**Authors:** Rosa Maria Carvalho, Fernanda Rocha Rodrigues Da Silva, Nayara Carvalho Goretti, Beatriz Julião Aarestrup, Fernando Monteiro Aarestrup

**Affiliations:** 1 Universidade Federal De Juiz De Fora, Brazil

## Background

Asthma is a lower airways inflammatory disease, often associated with rhinitis, which affects a significant number of teenagers and has impact on quality of life (QOL) .Exhaled nitric oxide levels (FeNO) evaluation is considered to assess the degree of airways inflammation. We aim to investigate in asthmatic teenagers the relationship between FeNO levels and QOL.

## Methods

Twenty-seven teenagers with asthma detected through the International Study of Asthma and Allergies in Childhood (ISAAC) questionnaire answered the ISAAC rhinitis module and underwent spirometry, FeNO measurement and evaluation of QOL through Peadiatric Asthma Quality of Life Questionnaire (PAQLQ). Student t test and Kolmogorov – Smirnov test were applied for means comparison; association measures were performed through Pearson and Spearman correlation tests; a p-value<0.05 was considered to be statistically significant.

## Results

23 asthmatic teenagers presented associated rhinitis (AR group) and four had only asthma (A group). Ventilatory function showed to be within the normal limits; mean (± standard deviation) values for FeNO were 50.67 (± 39.17) ppb, 5.02 (± 1.08) for PAQLQ total score and 4.87 (± 1.34) for "symptoms", 4.30 (± 1.56) for “emotional” and 5.67 (± 1.56) for “physical limitation" PAQLQ domains; there was no difference for FeNO values between AR and A groups. Association between FeNO and PAQLQ scores was observed for the total score (r=-0.47, p=0.01), "symptoms" (r=-45, p=0.02) and "emotional aspect" (r=-0.45, p=0.02) domains. The “symptoms" and “physical impairment” domains scores were lower in RA group and in those teenagers who had FeNO above 25ppb (p<0.05).

## Conclusions

For asthmatic teenagers evaluated, QOL impairment showed to be related to higher levels of FeNO and to association of asthma with rhinitis.

